# Interaction of *Streptococcus agalactiae* and Cellular Innate Immunity in Colonization and Disease

**DOI:** 10.3389/fimmu.2014.00519

**Published:** 2014-10-29

**Authors:** Sybille Landwehr-Kenzel, Philipp Henneke

**Affiliations:** ^1^Berlin-Brandenburg Center for Regenerative Therapies, Charité University Medicine Berlin, Berlin, Germany; ^2^Berlin-Brandenburg School for Regenerative Therapies, Charité University Medicine Berlin, Berlin, Germany; ^3^Department of Pediatric Pulmonology and Immunology, Charité University Medicine Berlin, Berlin, Germany; ^4^Center for Pediatrics and Adolescent Medicine, University Medical Center Freiburg, Freiburg, Germany; ^5^Center for Chronic Immunodeficiency, University Medical Center Freiburg, Freiburg, Germany

**Keywords:** *S. agalactiae*, cellular innate immunity, intestinal microbiota, colonization, invasion, sepsis

## Abstract

*Streptococcus agalactiae (Group B streptococcus, GBS)* is highly adapted to humans, where it is a normal constituent of the intestinal and vaginal flora. Yet, GBS has highly invasive potential and causes excessive inflammation, sepsis, and death at the beginning of life, in the elderly and in diabetic patients. Thus, GBS is a model pathobiont that thrives in the healthy host, but has not lost its potential virulence during coevolution with mankind. It remains incompletely understood how the innate immune system contains GBS in the natural niches, the intestinal and genital tracts, and which molecular events underlie breakdown of mucocutaneous resistance. Newborn infants between days 7 and 90 of life are at risk of a particularly striking sepsis manifestation (late-onset disease), where the transition from colonization to invasion and dissemination, and thus from health to severe sepsis is typically fulminant and not predictable. The great majority of late-onset sepsis cases are caused by one clone, GBS ST17, which expresses HvgA as a signature virulence factor and adhesin. In mice, HvgA promotes the crossing of both the mucosal and the blood–brain barrier. Expression levels of HvgA and other GBS virulence factors, such as pili and toxins, are regulated by the upstream two-component control system CovR/S. This in turn is modulated by acidic epithelial pH, high glucose levels, and during the passage through the mouse intestine. After invasion, GBS has the ability to subvert innate immunity by mechanisms like glycerinaldehyde-3-phosphate-dehydrogenase-dependent induction of IL-10 and β-protein binding to the inhibitory phagocyte receptors sialic acid binding immunoglobulin-like lectin 5 and 14. On the host side, sensing of GBS nucleic acids and lipopeptides by both Toll-like receptors and the inflammasome appears to be critical for host resistance against GBS. Yet, comprehensive models on the interplay between GBS and human immune cells at the colonizing site are just emerging.

## Introduction

*Streptococcus agalactiae*, Group B *Streptococcus* (GBS), is a commensal of the human intestinal and vaginal tract in 15–30% of healthy adults, but remains one of the most important invasive pathogens in newborn infants and the elderly (1–4). Every 10th neonate acquires GBS vertically during passage through the birth canal or shortly thereafter. In most cases, GBS can be assumed to become a normal constituent of the child’s microbiome. In other cases, other colonizing bacteria that expand on the expense of GBS may replace it. These dynamic processes early in life are generally safe, as 99% of at least temporarily colonized infants will never develop invasive GBS disease (5–8). The achievement of establishing a microbiome including GBS can best be judged in light of GBS late-onset diseases (LOD) and meningitis: if GBS does not adapt, it may cause fulminant disease. Notably, crossing the intestinal barrier and the blood barrier seems to be mechanistically linked. GBS serotype III, a particularly frequent isolate in neonatal meningitis, has recently been found to exhibit specific neurotropism through expression of the adhesion factor HvgA (9, 10). HvgA efficiently supports bacterial adhesion and transfer through to the intestinal wall and later across the blood–brain barrier, specifically the vascular endothelium and the choroid plexus (9). In other words, protection of the neonatal brain from GBS starts in the gut. Long-term neurological impairment of variable degree, which affects about 35–50% of infants surviving meningitis, has become particularly important in the Western world, where improvements in intensive care have decreased lethality of GBS invasive disease below 10% (9, 11).

By definition, GBS is a normal constituent of the “*intestinal microbiota*,” which comprises numerous bacteria, fungi, and protozoa. In a normal adult, the microbiota comprises more than 10,000 species, adding up to 1500 g in biomass. The composition of the microbiota is unique to the host and can be viewed as a personal fingerprint that emerges in early infancy (12, 13). Members of the bacterial phyla *Bacteroidetes, Firmicutes* (e.g., *Lactobacillus and Clostridum spp*), *Proteobacteria, Actinobacteria (*e.g., *Bifidobacterium spp.), Fusobacteria*, and *Spirochaetes* are the most important constituents of the gastrointestinal flora (12, 14, 15). Notably, the microbial composition underlies temporal changes during the first year of life and differs between children born vaginally and by caesarian section (16–18). A modulating role of breast milk feeding in the composition of the neonatal microbiome, e.g., by maternal secretory IgA, antimicrobial peptides, lactoferrin, and sCD14, has been shown by several investigators (19–25). In twins, it appears that both genetic and environmental factors contribute to the composition of the intestinal microbiota (22, 26, 27).

Colonizers of the human intestine are generally considered as “symbionts” that stabilize the intestinal homeostasis by acidification of the intraluminal milieu, maintenance of the transepithelial resistance, prevention of pathogen adherence, and continuous immune stimulation (28–32). Pathobionts are potentially pathogenic colonizers that usually reside in the intestine in coexistence with the host, but can cause severe local or systemic disease. In newborn infants, the most important examples are *E. coli*, enterococci, and GBS. The Janus face of GBS is underlined by the fact that 10% of all neonates are at least temporarily colonized by GBS (1–4), yet only 1% of these develops invasive disease (5–8). Beginning at birth, GBS colonization rates continuously increase to 20–30% in adults (1–4). This indicates that GBS transmission occurs not only perinatally, but also horizontally later in life. Next to the exposure to various bacterial strains, external factors like stress, drugs, diet, gastrointestinal infections, and endogenous factors, like diabetes, alter the composition of the intestinal microbiota (21, 33). These individual life style and health factors may explain in part, why GBS is an important cause of soft-tissue and urinary tract infections, arthritis, and sepsis in patients >65 years and those with chronic diseases such as stroke (OR 3.5), diabetes (OR 3.0), kidney or liver (OR 9.7) failure, and cancer (breast cancer OR 4.0) (34). In healthy adults, GBS predominantly colonizes the outer mucus layer of the colon, yet may occasionally reside in the small intestine as well (35, 36). In pregnant women, GBS is a frequent cause of urinary and upper genital tract infections, intra-amniotic infections, and sepsis (37, 38). Whether GBS colonization usually, i.e., in infants as well as adults, starts in the intestinal tract and expands from there to other sites, or whether GBS subsets develop at independent colonization sites of the same human influenced by the site-specific microenvironment, has not yet been clarified. Accordingly, the origin of differences in serotype distribution between non-pregnant adults with invasive disease, where serotype V contributes to almost 30% of cases, and pregnant women or newborn infants, where serotypes III and Ia predominate, is not well understood (7, 39, 40).

## The Pathogen Site: GBS Virulence Strategies to Progress from Colonization to Disease

What can we learn from neonatal GBS sepsis models with respect to factors regulating colonization and invasion? GBS sepsis in newborn infants can be divided into early-onset disease (EOD), which occurs within the first week of life by vertical GBS transmission during delivery, and LOD, which occurs through vertical or horizontal transfer and manifests between day 7 and 3 months of age (41). However, since more than 90% of all EOD cases manifest as sepsis, pneumonia, or meningitis within the first 24–48 h of life, some authors limit early-onset sepsis to the first 72 h after birth (40, 42). Peripartum antibiotic prophylaxis has markedly decreased the incidence and case fatality rate of EOD; the incidence or clinical course of LOD, however, has remained largely unaffected. The clinical picture of LOD typically manifests as hyperinflammatory syndrome with meningitis. As outlined above, GBS serotype III is greatly overrepresented in LOD, with clonotype ST17 contributing up to 90% of all clinical isolates found in meningitis (as compared to colonizing strains). This led to the phenotypic description of GBS III ST17 as hypervirulent clone (43–47). A recently published longitudinal study found that in LOD, GBS and other bacteria isolated from the blood of preterm infants genetically matched clones previously found in the patients’ stool (48). Accordingly, in many cases, LOD likely results from systemic spread of intestinal GBS, and not directly from vertical or horizontal transmission. Nevertheless, postnatal exposure with maternal GBS III ST17, e.g., via breast milk, appears to underlie some sepsis cases (49).

For the development of sepsis by GBS spreading from the intestine, the bacterium has to undertake three consecutive steps: (i) colonization of the colon and potentially the small intestine, (ii) translocation across the intestinal epithelium, and (iii) immune evasion preventing GBS clearance from the blood stream (summarized in Table 1).

**Table 1:** **GBS virulence factors and their role in transition from colonization to invasive disease**.

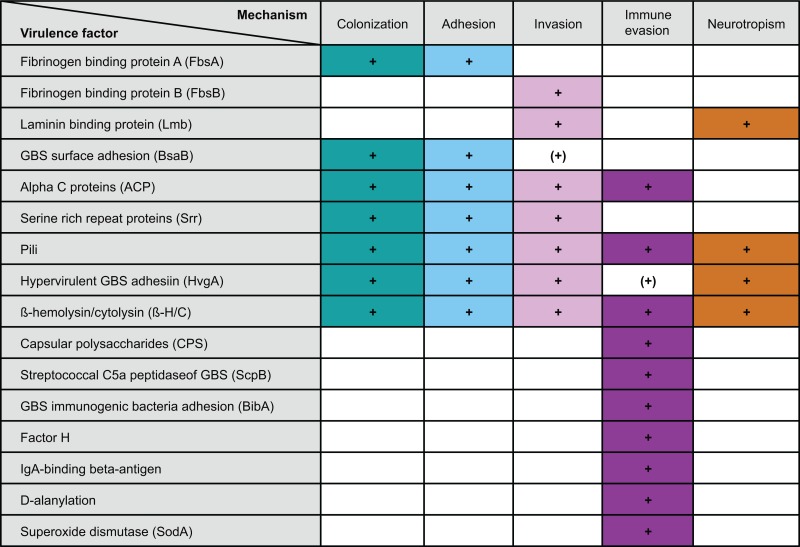

The first and pivotal step in GBS colonization is adhesion. Adhesion factors are expressed on the bacterial surface and allow GBS to bind to extracellular matrix proteins and epithelial cells of the colon and the genital tract resulting in biofilm formation (50, 51). Adhesion factors can additionally promote invasion, either by disruption of the epithelial cell layer or by modulation of the epithelial cytoskeleton and the junctional protein assembly, which in turn allows for paracellular translocation (52–55).

Two adhesion factors mediate attachment to the extracellular matrix. They are named according to their specific ligand: (i) fibrinogen-binding proteins and (ii) laminin-binding proteins (Lmb). While fibrinogen-binding protein A (FbsA) promotes adhesion, fibrinogen-binding-protein B (FbsB) mediates GBS invasion into host cells. FbsA and FbsB bind to both immobilized and soluble fibrinogen (56, 57). Bacterial attachment to extracellular matrix via Lmb seems important for GBS translocation across the intestinal epithelium and the blood–brain barrier (58, 59). Furthermore, the GBS surface adhesin BsaB binds to fibronectin (60). However, whether BsaB contributes to GBS invasion or mainly promotes colonization with GBS via its biofilm enhancing effects has not been fully resolved, yet.

Another group of GBS adhesion factors is characterized by the highly conserved LPxTG (Leu–Pro–X–Thr–Gly) motif at the C-terminus (61). GBS LPxTG is cleaved between Thr and Gly by the transpeptidase Sortase A, which covalently binds GBS to the cell wall and thus promotes both colonization and invasion (62). The first group of the LPxTG containing adhesion factors is the family of Alpha C proteins, which are encoded by the *bca* (group B, C protein alpha) gene and expressed on most strains of serotype Ia, Ib, and II (63). Alpha C proteins are further characterized by long tandem repeating elements, which allow antigenic variations, and a conserved N-terminal domain, encoding 185 amino acids (61, 64–67). GBS isolated from mothers who recently delivered a child with invasive GBS disease show increased expression of alpha C protein tandem repeats (“aa”). This is associated with increased susceptibility to opsonophagocytic antibody-mediated killing as compared to the GBS isolated previously from the respective newborn infant with sepsis (67). In line with these observations, low tandem repeat expression during infections seems to impair the specific antibody response and antibody-mediated killing (61, 64, 65, 68, 69). Furthermore, GBS alpha C proteins can promote invasion of human epithelial cells via α1β1-integrin binding (66).

The second group of LPxTG containing adhesion factors are serine-rich repeat (Srr) proteins, which interact with human keratin (Srr-1) and which enhance GBS virulence in mice (Srr-2) (70). Only recently, fibrinogen has been identified as Srr binding partner (71). Interestingly, while highly virulent serotype III strains express Srr-2 (70, 71), other GBS strains express Srr-1 (serotype Ia, Ib, Ic, II, and V).

Additional structures involved in adhesion are Pili, which were first described in GBS in 2005 (72). Pili promote colonization of epithelial cell surfaces, support biofilm formation, and facilitate translocation across the blood–brain barrier (51). They consist of a major shaft subunit, the backbone protein BP, which is critical for pilus assembly, and the two ancillary proteins AP1 and AP2 (73). Three pilus islands (PI1, PI2a, and PI2b) were identified. In mice, PI2a is essential for GBS virulence (74), and pilus island specific antibodies enhance opsonophagocytic killing and protect from sepsis (75, 76). Yet, development of pilus structures as vaccine candidates was hampered by variable pilus expression in GBS (75, 76).

Comparative expression analysis between clones with different clinical virulence (based on the disease phenotype in infants) led to the identification of the surface-anchored hypervirulent GBS adhesin (HvgA) as a specific virulence factor in GBS ST17 (9). Similar to pili, HvgA mediates both colonization and invasion in the intestine and confers meningeal tropism in neonatal mice (9, 10). Interestingly, GBS isolated from blood and cerebrospinal fluid during invasive disease express higher HvgA levels as compared to GBS cultured *in vitro*, indicating upregulation of HvgA expression during infection (9). Moreover, the ability of GBS ST17 to spread from the intestinal lumen is linked to the age of the mice, since 60–70% of preweaning mice (15–21 days old) succumb after enteral infection with *hvgA*-expressing GBS, whereas mice ≥4 weeks are protected (9). An experiment of nature, which we recently observed, suggests that yet to be identified changes in GBS virulence likely contribute to sepsis onset. An HvgA-positive strain of GBS, which was transmitted probably through breast milk from the mother, induced two episodes of sepsis each in twins, i.e., a total of four episodes, in a synchronous fashion. In all cases, sepsis started with soft-tissue infections of the lower oral cavity border, which implies a temporarily highly invasive and reproducible behavior of GBS (49).

Group B *Streptococcus* translocation across the epithelial barrier is further facilitated by the virulence factor β-hemolysin/cytolysin (β-H/C) (77–79). β-H/C induces cytolysis in eukaryotic cells and promotes bacterial invasion across epithelial and endothelial walls, including the blood–brain barrier. In mice, β-H/C induces placental inflammation and preterm birth, independently of bacterial ascension (80). β-H/C-deficient GBS show impaired virulence in various *in vivo* models including pneumonia, sepsis, and meningitis (77, 79, 81). However, at sublytic concentrations, β-H/C drives expression of the anti-inflammatory cytokine IL-10 and inhibits both IL-12 and NOS2 expression in GBS-infected macrophages (82). Thus expression levels of β-H/C appear to determine whether GBS stabilizes its niche to allow for colonization, or whether GBS becomes invasive. Additionally, the pore-forming toxin and co-hemolysin CAMP factor may contribute to GBS pathogenesis under certain circumstances (83, 84), but is dispensable for systemic virulence (83–86).

Distinct types of capsular polysaccharides (CPS), which underlie the serotyping system, allow for immune evasion, since α2 → 3 linked sialic acid modifications use molecular mimicry with host sugar epitopes. Direct binding to immunoglobulin-like lectins (Siglecs) on leukocytes inhibits complement C3 activation on the bacterial surface (87–89). Complement inactivation is further supported by ScpB (Streptococcal C5a peptidase of GBS)-mediated proteolytic C5a inactivation (90) and reduced complement binding through the inhibitory factors BibA (GBS immunogenic bacterial adhesin) (91) and factor H (92). Immunoglobulin binding, an essential precondition for opsonophagocytosis, is hampered by the IgA-binding beta-antigen of the c protein complex, which recognizes the Fc region of human immunoglobulin A (93). GBS evades phagolysosomal processing by neutralizing reactive oxygen species via BibA, β-H/C, superoxide dismutase (SodA), and additional unknown factors (77, 78, 91, 94–96). Similarly, binding of intestinal and circulating antimicrobial peptides can be suppressed by intrinsic GBS mechanisms such as d-alanylation of lipteichoic acid, which is catalyzed by the *dlt* operon. Down-modulation of d-alanylation decreases the negative surface charge, which in turn is important for cationic binding of antimicrobial peptides such as colistin (97).

## Regulation of GBS Virulence

As outlined above, the human intestine is the natural niche for GBS. In other words, GBS thrives in healthy hosts. Accordingly, it is for the benefit of both GBS and the host if GBS is kept in a colonization state and does not exhibit its aggressive traits. Thus, expression of virulence factors in GBS must be tightly regulated. Two-component control systems, which typically consist of a membrane-linked histidine kinase sensor and a cytoplasmatic transcriptional element, are common in bacteria and more than 4000 regulatory systems have been described (98, 99). In GBS, CovS/CovR (Control of virulence Sensor/Regulator) tightly regulates the expression of pili, BsaB, *hvgA, cyIE*, which is involved in β-H/C expression, and many other genes (60, 100, 101). CovS acts as a pH sensor. In an acidic milieu, such as the vagina, CovS remains in an autophosphorylated state and activates the regulatory domain CovR by transphosphorylation at the aspartate residue D53. Subsequently, the phosphorylated form of CovR acts as a repressor. An increase in pH reduces CovS phosphorylation and induces the expression of β-H/C (102). In addition to pH, high glucose levels, peripheral insulin resistance, and passage through the intestine modulate CovR/CovS in mice (103–105). Inactivation of the CovR system increases GBS adherence to epithelial cells (106) by upregulation of PI1 expression (104). In line with this, GBS mutant in either CovS or CovR show increased hemolysis and approximately 80-fold upregulated HvgA (9).

Next to CovS, the serine/threonine kinase Stk1 acts as a sensor for environmental stimuli. Stk1 activation leads to phosphorylation of CovR at threonine 65. GBS with Stk1 mutations show reduced expression of β-H/C; susceptibility to opsonophagocytic killing and oxidative stress is increased (107, 108). Thus, while CovR phosphorylation at the aspartate residue D53 by CovS leads to protein activation associated with reduced virulence, Stk1-mediated phosphorylation at the threonine residue T63 increases the expression of β-H/C (108). Abx1, an additional partner within the CovS/CovR system, displays complex counterbalancing activity (107–109). As transmembrane protein Abx1 holds direct proximity to CovS, which it partially antagonizes. Both functional loss and overexpression of Abx1 hampers GBS virulence expression, and Abx1 expression itself is critically dependent on functional β-H/C formation (109). This system “fine tunes” GBS and may explain, at least in part, the loss of virulence of hyperhemolytic mutants due to CovR inactivation or Abx1 overexpression.

In summary, the CovS/CovR system inhabits a central role in the control of GBS virulence. It is tightly regulated, and specific environmental factors allow for subtle adaptation of the bacterial phenotype to the site of colonization/infection.

## The Host Site: Toll-Like Receptors and the Inflammasome Mediate Host Resistance and Fatal Inflammation

Neonatal mice are exquisitely sensitive for GBS. Less than 100 bacteria injected *s.c*. constitute the lethal dose 90%. Under these conditions, disease is at least partially immune mediated, since the inflammatory cytokine TNF alone accounts for approximately half of the deaths (110). An important ligand receptor interaction resulting in TNF formation is that between diacylated lipoproteins and Toll-like receptors (TLR)2/6 heterodimers (111). Deletion of the diacyl transferase *Lgt* from GBS results in the same change in GBS sepsis phenotype as that observed in conventional TLR2 knock-out mice. The TLR-dependent recognition of bacterial lipopeptides can be assumed to primarily occur on the cell surface (112), although endosomal recognition of lipopeptides has recently been demonstrated (113). Next to lipoproteins, nucleic acids from GBS potently activate inflammatory genes in phagocytes. At least three distinct signaling pathways engaged by GBS nucleic acids have been identified. First, intracellular recognition of GBS DNA by one or several currently unknown cytosolic receptors results in the formation of type I interferons in a TBK1 and IRF3-dependent fashion (114) (IFN pathway). Second, two pathways involve recognition of GBS RNA. The first engages an UNC-93B- and MyD88-dependent pathway (115, 116) (TLR pathway), which has been shown to utilize TLR7 in dendritic cells (117), whereas the cognate TLR in macrophages is still elusive (116). The second pathway involves cytosolic sensing of RNA and β-hemolysin through the intracellular NLRP3 inflammasome (NOD-like receptor family, pyrin domain containing 3), which mediates IL-1β maturation in macrophages and dentritic cells (118, 119). Accordingly, in a mouse GBS sepsis model, NLRP3-deficiency is associated with significantly increased lethality (118, 119). It appears that NLRP3 and GBS RNA closely interact in the cytosol, and disruption of the GBS bearing phagosomes allows NLRP3 and GBS RNA to get into close contact (118). Discrimination of bacterial RNA from human ribosomal and transfer RNA appears to depend at least partially on the RNA methylation status (120). In *S. aureus*, methylated ribosomal RNA is recognized by TLR13, a TLR that currently lacks a human homolog. Accordingly, *S. aureus* strains that have acquired the methylase erm, conferring erythromycin resistance, have lost their TLR13-activating potential (121).

In summary, sensing of GBS lipoproteins and nucleic acids mediates activation of macrophages and dendritic cells and contributes to resistance against GBS and disease progression during sepsis. Yet, the contribution of these systems to containing GBS at mucosal sites is currently unclear.

## IL-10 and Its Role in GBS Colonization and Disease

**Figure 1:**
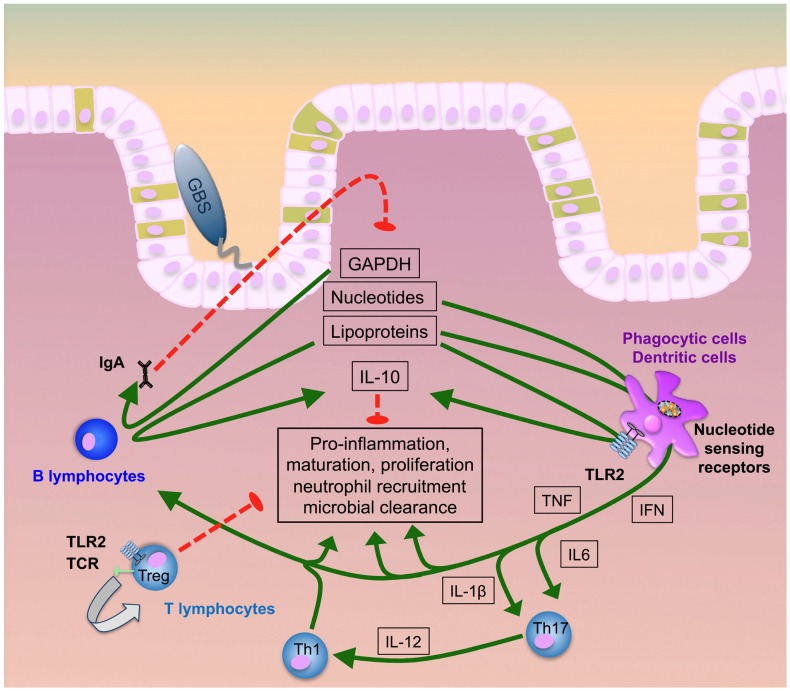
**GBS transition to invasive disease**. GBS lipoproteins, GAPDH, and nucleic acids majorly contribute to intestinal immune activation inducing pro-inflammatory responses, neutrophil recruitment, cellular proliferation, maturation and, finally, microbial clearance. Early induction of specific antibody release by B plasma cells and IL-10 secretion by both B-lymphocytes and phagocytes act as a negative feedback loop to counter-regulate hyperinflammation. Uncontrolled increase of IL-10, in contrast, hampers neutrophil recruitment and bacterial elimination. At the same time, T-lymphocytes can directly interact with GBS ligands or receive pro-inflammatory signals via monocytic cytokines. While Th1 and Th17 cells promote infection control, activation of regulatory T cells prevents hyperinflammation and supports post-infectious healing.

Very recently, the glycolytic enzyme glycerinaldehyde-3-phosphate-dehydrogenase (GAPDH) from GBS was shown to subvert immunity by inducing IL-10 (103, 122, 123). This adds to the kaleidoscope of functions of an enzyme, which is largely known for its role in bacterial energy generation (124–127) (summarized in Figure 1). GBS GAPDH is expressed as a surface molecule and in a soluble form (126, 128, 129). In mice, it directly activates B- and, to a lower extent, T-lymphocytes and induces the maturation of anti-GAPDH Ig-secreting plasma cells (130). Both endogenous and systemically administered GAPDH specific antibodies significantly reduce intestinal GBS colonization and protect from invasion of GAPDH-expression GBS strains. On the other hand, administration of recombinant GAPDH induces systemic release of IL-10 in mice and increases susceptibility to invasive GBS disease and bacterial invasion of *per se* non-invasive strains (82, 122, 130, 131). In line with these observations, IL-10-deficient mice are protected from invasive GBS disease and show improved survival in various sepsis models (122, 131–133). These effects are further consistent with the observation that reduced IL-10 activity increases neutrophil recruitment and bacterial killing (122, 130, 131). However, in earlier investigations, IL-10 given before infection protected neonatal mice from GBS sepsis and correlated with TNF suppression and improved survival (134). This effect was strictly dependent on the time of IL-10 application, since simultaneous or post-infectious administration failed to mediate protective effects. In contrast, in LPS-TLR4 induced septic shock models IL-10 improved survival even when given simultaneously with or early after LPS administration (133, 135, 136). Monocytes, macrophages, and T- and B-lymphocytes are all sources of IL-10 (Figure 1). IL-10 induction in macrophages depends on JNK signaling, which in turn is critical for the outcome in GBS sepsis (137–140). Expression of IL-10R (IL-10 receptor) and TGFBR1/TGFBR2 (TGF-beta receptors 1 and 2) and the presence of their specific ligands are pivotal to preserve immune homeostasis (141–144). *In vitro*, IL-10 reduces NF-kB signaling and the expression of TLRs, MyD88, and TIR in resident intestinal macrophages (iM) and circulating monocytes (145). In neonatal mice, the inflammatory program of iM is shifted toward IL-10 as the signature effector molecule via TGF-beta as a signaling intermediate (146). Patients with defects in the IL-10 or IL-10R gene develop severe, early-onset inflammatory bowel disease. Thus IL-10 is critical for intestinal inflammation control (147–149). Since, mice with a targeted deletion of STAT3 in macrophages and neutrophils (LysMcre/Stat3^flox/−^) develop spontaneous enterocolitis, it seems that tissue macrophages constitute the major source of IL-10 (150). Macrophage expression of IL-10 is dependent on TLR-dependent sensing of the microbiota and signal transduction by the adapter protein MyD88 (151). The multitude of functions of IL-10, its site-specific functions and the dynamics in its inducible effects all contribute to its highly complex role in stabilizing the interface between GBS and host.

## GBS in (Transient) Immunodeficiency

The so-called human model, where patients with monogenetic variations provide clues for the role of cell specific immunity, has been powerful in better understanding host–microbe interactions leading to coexistence and disease. An intriguing example is MyD88/IRAK-4-deficiency with its specific susceptibility to staphylococci, streptococci, and *Pseudomonas spp*. However, the search for monogenetic immunodeficiency disorders underlying susceptibility to invasive GBS infections has only been partially successful so far. One patient with very late-onset GBS sepsis suffering from IRAK-4 deficiency has been reported, supporting that cellular innate immunity and the TLR system are important for resistance against GBS (152–154). It can be expected that whole exome or genome analysis in families with GBS sepsis will provide further information in this regard in the future.

Next to the genetic absence of specific factors in cellular innate immunity, transient changes in innate immunity may underlie susceptibility to invasive GBS disease in newborn infants. Neonatal sepsis is characterized by excessive inflammation, since high levels of pro-inflammatory cytokines can be measured in the serum of newborn infants suffering from invasive GBS disease. *In vitro*, PBMC from healthy neonates rapidly respond to GBS with the formation of large amounts of IL-6, TNF, IL-8, and IL-1β (155–157). Since insulin suppresses the cytokine formation in response to GBS, peripheral insulin resistance present in newborn infants and particularly during sepsis may promote the inflammatory process (158). Excessive stimulation of immune cells may be further enhanced by a reduction of antimicrobial phagocyte properties, which are markedly impaired in neonates and may allow for pathogen persistence and failure to contract the immune response (159–161). GBS persistence likely results from reduced G-actin polarization and L-selectin expression in newborn, especially preterm infants, which results in impaired neutrophil adhesion and migration (162, 163). Moreover, low expression of opsonizing complement components, immunoglobulins, reactive oxygen species, the integrin CD11b/CD18, and Fc-gamma receptors II and III (164–166) and a predominant polarization of T-lymphocytes toward Th2 and Th17 may overall interfere with timely and rigorous clearance of invasive GBS (161, 167–173). How can the high levels of IL-10 in neonatal GBS infections, as observed in mice (174–177) and humans (178–180), be explained in view of the generally highly inflammatory state? Although conclusive evidence on this matter is lacking, it is tempting to speculate that the negative impact of IL-10 on neutrophil function, which allows for pathogen expansion, overrides the direct anti-inflammatory properties of IL-10 on the mononuclear phagocyte system. In this scenario, the net result in neonatal GBS sepsis is inflammatory despite high IL-10 levels.

## Conclusion

Group B *Streptococcus* comprises several regulatory systems that respond to the microenvironment and, by steering adhesion and virulence factors, allow for colonization of mucosal niches in the genital and (lower) intestinal tract. Colonization is further propagated by the GBS-intrinsic ability to manipulate local cellular immunity. Yet, under only partially understood conditions GBS looses its colonizing trait and invades the host. Then, immune mechanisms that usually stabilize the natural GBS niche may lead to detrimental immunopathology. Rather subtle changes on the single immune cell level in newborn infants appear to facilitate the escalation from a beneficial site-specific response to sepsis and meningitis. Better understanding of the dynamic expression of virulence traits in GBS, and of the cellular immunology that shapes the GBS niche, will hopefully pave the way for preventing livelong disabilities inflicted by a normal component of the microbiota.

## Conflict of Interest Statement

The authors declare that the research was conducted in the absence of any commercial or financial relationships that could be construed as a potential conflict of interest.
